# Mixed Method Study to Explore Ethical Dilemmas and Health Care Workers' Willingness to Work Amid COVID-19 Pandemic in Palestine

**DOI:** 10.3389/fmed.2020.576820

**Published:** 2021-01-05

**Authors:** Beesan Maraqa, Zaher Nazzal, Therese Zink

**Affiliations:** ^1^Primary Health Directorate, Ministry of Health, Ramallah, Palestine; ^2^Family Medicine Residency Program, Faculty of Graduate Studies, An-Najah National University, Nablus, Palestine; ^3^Department of Family and Community Medicine, Faculty of Medicine and Health Sciences, An-Najah National University, Nablus, Palestine; ^4^Department of Family Medicine & School of Public Health, Brown University, Providence, RI, United States

**Keywords:** COVID 19, ethical dilemmas, willingness to work, duty to work, health care workers, Palestine

## Abstract

**Background:** The high potential risks involved in working in a healthcare setting during a pandemic and the associated fear that may affect health care workers' (HCWs') willingness to work are important to understand to eliminate potential barriers to working. This study aimed to assess Palestinian HCWs' willingness to work and the related factors as well as to explore their ethical dilemmas during the coronavirus disease 2019 (COVID-19) pandemic.

**Materials and Methods:** Quantitative (survey questionnaire) and qualitative (semi-structured interviews) data were collected. Frontline HCWs (*n* = 550) received an online survey link via closed institutional networks. Frequencies summarized the data, and chi-square compared variables and outcomes. Odds ratios (ORs) and multivariable analysis examined predictors for willingness to work. Fifteen HCWs (physicians, nurses, and lab and radiology technicians) were purposefully sampled and agreed to interviews to explore their thoughts, motivations, and worries. Thematic analysis focused on ethical dilemmas to enhance the breadth and the depth of the study.

**Results:** Almost 25% of surveyed HCWs were not willing to work during the pandemic. Logistic model results showed that physicians and nurses had higher willingness to work than others (*p* = 0.004, Adj. OR = 3.5). Lower stress levels and longer professional experience were predictors of more willing to work (*p* = 0.03, Adj. OR = 2.5; *p* = 0.03, Adj. OR = 2.6, respectively). Interviews showed that willingness to work did not preclude HCWs from fulfilling their duties despite grueling workloads and grave fears about safety and security. HCWs felt poorly prepared, unappreciated, and frustrated by unfair work distribution. The occupation presented additional safety issues.

**Conclusion:** Physicians and nurses were more likely to comply with a commitment to their professional ethics and the duty or obligation to work. Stress levels could be mitigated in the future with better leadership, adding supports to address mental health and psychosocial challenges to enhance HCWs' well-being and improve quality of care. The realities of the occupation added additional threats and uncertainty.

## Introduction

The coronavirus disease pandemic of 2019 (COVID-19) was an unprecedented challenge for health care systems across the globe. Frontline health care workers (HCWs) were in the midst of contradictory and limited information about the type, severity, infectiousness, and necessary precautions required during the outbreak.

The COVID-19 pandemic with the rapid spread especially in Europe and United States (US) caused significant concerns as to how best to provide health care in emergency conditions and scarcity of resources (1, 2). The need goes even deeper in the Middle East and North Africa region (MENA), where the existing infrastructure is under stress, which likely intensifies the uncertainty and widens the gaps between those with more robust digital capability and those without (3).

Palestine is one of the countries struggling with compounding challenges of uncertainty, fragility, social mobility, and poverty. In addition, the pandemic reveals “triple tragedies,” composed of the COVID-19 pandemic, the politics of continued occupation by Israel, and the challenge of Intra-Palestinian dissent (4). The health care system in Palestine is divided into three levels: primary, secondary, and tertiary. The primary level represents the gateway into the health care system, and the secondary and tertiary consist of hospitals and rehabilitation centers. The four main health service providers working in the Palestinian Territories are the Ministry of Health (MOH), United Nations Relief and Works Agency (UNRWA), non-governmental organizations (NGOs), and the private sector. The key providers of primary care services are MOH and UNRWA. The primary suppliers of secondary services are the MOH and NGOs. The private sector is the largest source of tertiary care. For this pandemic, the major workload was on primary health care (PHC) workers who had to trace contacts and screen high-risk groups and emergency departments at major hospitals, in addition to newly established COVID-19 hospitals that added a challenge for the scarce PHC personnel and resources. It is believed that those delivering health care have a strong obligation to perform, often in the face of personal danger—a duty that is enshrined in the professional codes of conduct (5). However, any emergency event involving contagion or contamination, as with the COVID-19 pandemic, has the potential to alter HCWs' willingness to work for different reasons (6). A recent Cochrane review of previous pandemics reported lack of training about the infection itself and how to use personal protective equipment (PPE), shortage of and low-quality PPE supplies, as well as increased workloads and fatigue among HCWs, ambiguous work settings, and rapidly changing guidelines as the tip of the iceberg during prior experiences, whereas HCWs' fear of catching infection themselves or infecting their families and the psychosocial burden of the pandemic were hidden below the surface (7).

In severe acute respiratory syndrome (SARS) outbreak on 2003, frontline HCWs found themselves in the midst of conflicting and confusing reports and reflected ethical issues such as trust, truth-telling and relationships with colleagues, resource allocation, and public health and infection control (8). Major ethical dilemmas that HCWs could face during this pandemic are balancing their ethical duty to care for their patients against their concerns of contracting COVID-19 and spreading it to their patients and families. Limited availability of PPEs, inequitable distribution of available equipment, and limited and constantly changing recommendations could increase such concerns (9).

Regarding the COVID 19 pandemic, a variety of critical ethical concerns arising from fair allocation of scarce medical resources such as ventilators and resuscitation services (10, 11) to challenges facing HCWs during their duty to treat in extreme circumstances is recognized (12). Cross infection worries place HCWs at a challenging intersection in their duty to work whether to relieve themselves of their work duties if possible or to respond to the ethical sense of duty to patients and community (13). However, studies addressing ethical problems are scarce in the Eastern Mediterranean region, where the trend of mortality and morbidity in COVID-19 varies from that of the European and American regions.

Taking into consideration the potential risks involved in working in a health care setting during a pandemic, and the associated fears, it is important to explore how motivated HCWs are to continue to work during such a crisis and what factors might influence their decisions (14). HCWs' willingness to work in a pandemic ranged from 23.1% at Hong Kong's influenza A (H1N1) pandemic in 2009 to 95.8% in US medical students targeting a hypothetical influenza pandemic. Females were less willing and able to work than males. By working group, physicians were more likely to be willing to work, followed by nurses and other HCWs. Personal safety at work and perception of the risk of a pandemic have been described as factors influencing the willingness to work as well as the availability of PPE and previous training (15). Careful management of these factors can make it possible to implement strategies to address the concerns and fears of HCWs and to eliminate potential barriers to working. No existing literature on the willingness to work on COVID-19 pandemic has been identified. In addition, no one discussed the willingness to work related to ethical concerns. In this study, we aimed to assess Palestinian HCWs' willingness to work and the related factors. Additionally, we intended to explore the ethical dilemmas of concern during the COVID-19 pandemic.

## Materials and Methods

### Study Design

The research involved the combined use of qualitative (interviews) and quantitative (questionnaire) data collection to assess the willingness to work among frontline HCWs and contributing factors. Data were collected in two phases. First, a quantitative cross-sectional study using an online questionnaire that targeted frontline HCWs (physicians, nurses, and lab and radiology technicians) working in hospitals and PHC centers was utilized, and a second phase used semi-structured interviews to enhance the breadth and the depth of the study.

### Quantitative Phase

#### Data Collection and Sampling

A self-administered questionnaire was constructed and refined from previous studies (16, 17) to address the study objective. It was designed using the Web-based application Google Forms, then the questionnaire link was distributed to HCWs through closed institutional (WhatsApp) groups. This method takes advantage of the high rates of Internet use among Palestinians and allowed us to reach as many frontline HCWs as possible given the COVID-19 quarantine and social distancing guidelines. 2 weeks later, a follow-up reminder was sent to HCWs, and a final reminder was sent after another 2 weeks. The questionnaire was completed during the 2nd month of the COVID outbreak in Palestine. Respondent anonymity was preserved using the Web-based survey method for data collection and collation. Web-based tools (such as Google Forms) protect information confidentiality when returning the questionnaire and prohibit other participants from accessing information. Furthermore, no identifying questions were included in the survey.

Sample size calculations for the quantitative part were based on the formula: [Necessary Sample Size = Z^2^^*^ expected willingness prevalence ^*^ (1- expected willingness prevalence)/(margin of error)^2^]. Using an expected proportion of 50%, a 95% confidence interval (CI), and a 5% absolute precision on either side of the proportion, the minimum required sample size was 340 HCWs. This was inflated by 60% to compensate for the expected non-response rate and sent to 550 HCWs using a convenience sampling method.

#### Instrument

The questionnaire is composed of two parts. The first part assessed participants' basic demographic information: age, sex, experience, work setting (PHC vs. hospital), and having children. Whether or not they lived with family during the outbreak and dealt with positive COVID-19 cases was explored. Willingness to work was assessed using a direct yes/no question, “Are you willing to work during this COVID-19 pandemic?” The second part assessed their stress level, attitudes, and disappointments during their work duty amid the COVID-19 outbreak with a Likert scale of 0 to 5. A direct question has been asked about HCWs' feeling of stress during the pandemic “I feel stressed because of the COVID-19 outbreak,” and a group of questions have been asked about factors that may affect their stress, such as fear of being susceptible or transmitting the disease to their families and lack of experience and preparedness. A rank of more than three was used as a cut point (Supplementary Material 2).

The questionnaire was pretested for its validity and reliability. Three experts in the field reviewed the instrument for face and content validity, and we piloted it on 20 HCWs with similar sociodemographic and professional characteristics to the study population. This helped us reframe and reword some questions and provided feedback on the feasibility of the Google Forms questionnaire link. Reliability was measured by the internal consistency of the questionnaire with a Cronbach Alpha of 0.90, which indicates excellent reliability.

#### Statistical Analysis

Quantitative data analysis was completed with the Statistical Package for the Social Sciences software (SPSS version 20.0). Categorical sociodemographic data were summarized by frequencies and percentages of occurrence. The chi-square test was used to compare between categorical variable and the study outcome; associations are presented as odds ratios (ORs) and 95% confidence intervals (95% CIs). Multivariable analysis was conducted to assess for predictors of willingness to work and to control for confounders. A *p*-value of 0.05 was used to determine statistical significance.

### Qualitative Phase

#### Data Collection and Sampling

Second, a qualitative study using semi-structured interviews explored HCWs' thoughts, worries, fears, reasons, and motivations related to the duty to work during the pandemic. The interview guide was developed from literature review, and the preliminary knowledge of the quantitative findings allowed us to explore areas such as participant motivations in greater depth (13, 18, 19). Initial questions explored HCWs' thoughts about their duty to work during the COVID-19 pandemic, factors motivating them to work, how they perceived their relationships with their colleagues, the barriers they faced, and their most challenging issues. A final question probed their perceptions of the risks and fears about working in the current circumstances (Supplementary Material 1).

Fifteen frontline HCWs (physicians, nurses, and lab and radiology technicians) were purposively sampled (20). Interview participants were chosen for various geographical locations on the West Bank (North, Center and North), taking both gender and job requirements into account. They were approached toward the end of the third month of the COVID outbreak *via* e-mail or text. If the HCW agreed to be interviewed, informed consent was obtained verbally and confidentiality was affirmed. HCWs were interviewed in a private place of their choice. Interviews were conducted face-to-face when possible or by telephone to those working in quarantined areas. The interviews were audio recorded and lasted an average of 30 min.

#### Interview Analysis

Transcripts were transcribed word for word, reviewed against the transcripts in order to ensure accuracy, and translated into English. One researcher (TZ) sorted data into topical categories for further analysis and identification of patterns and themes and assigned codes. These were discussed with the interviewer/researcher (BM) and further organized into themes and subthemes with a focus on the different bioethical dilemmas participants faced. Discussion occurred until consensus was reached and appropriate quotes were selected. The analysis methods used are defined by Creswell and Poth (21).

### Ethics Statement

Ethical approval was secured from the institutional review board (IRB) at An-Najah National University. All participants were informed about the purpose of the study, the voluntary nature, and anonymity, and confidentiality was assured before they gave their consent.

## Results

### Quantitative Survey

We targeted 550 HCWs and received 400 filled questionnaires, a 73% response rate. However, 43 were incomplete, so we had 357 valid questionnaires. Of the respondents, 43.7 and 45.1% were physicians and nurses, respectively. The mean age was 36.7 years, and 52.8% were older than 35 years. More than half of participants were female (55.3%) and worked in PHC centers (56.9%). Most had children (73.6%) and lived with their families during the pandemic (89%). Thirty-six percent dealt directly with positive COVID-19 cases (Table 1).

**Table 1:** Participants' background characteristics and the association with willingness to work.

**Variable**	**Total (*n* = 357) *N* (%)**	**Willing to work**		*P-*value^*^
		**Yes (%)268 (75.1)**	**No (%) 89 (24.9)**	
Age				<0.001
<35 years	166 (47.2)	109 (56.7)	57 (34.3)	
≥35 years	186 (52.8)	154 (82.2)	32 (17.2)	
Sex				0.056
Female	197 (55.3)	140 (71.1)	57 (28.9)	
Male	159 (44.7)	124 (79.9)	32 (20.1)	
Work setting				0.036
PHC	203 (56.9)	161 (79.3)	42 (20.7)	
Hospital	154 (43.1)	107 (54.6)	47 (30.5)	
Job title				0.024
Physician	156 (43.7)	120 (76.9)	36 (23.1)	
Nurse	161 (45.1)	125 (77.6)	36 (22.4)	
Others	40 (11.2)	23 (57.5)	17 (42.5)	
Experience				<0.001
<10 years	154 (43.1)	98 (63.6)	56 (36.4)	
≥10 Years	203 (56.9)	170 (83.7)	33 (16.3)	
Having children				0.015
Yes	269 (73.6)	211 (78.4)	58 (21.6)	
No	87 (24.4)	57 (65.5)	30 (34.5)	
Living with family				0.48
Yes	316 (89)	235 (74.4)	81 (25.6)	
No	39 (11)	31 (79.5)	8 (20.5)	
Dealt with COVID-19 case				0.3
Yes	129 (36.1)	101 (78.3)	28(21.7)	
No	228 (63.9)	167 (73.2)	61 (26.8)	

* *Chi square test*.

One quarter of study participants (24.9%) were not willing to work during the pandemic. The results of the univariate analysis, elucidating associations with willingness to work during COVID-19 pandemic, are shown in Table 1. More than 80% of HCWs ≥35 years of age showed significantly higher willingness to work (*p*-value 0.001). PHC workers, physicians and nurses, were more willing to work during the pandemic with significance, *p*-value 0.036 and 0.024, respectively. Finally, 78% of those reported to have children had significantly higher willingness to work (*p*-value of 0.015).

HCWs' willingness to work in relation to their attitudes and other factors were assessed using the chi square test. Willingness to work was higher among HCWs who did not report stress about catching the infection and felt safe (*p* = 0.007 and 0.001, respectively). Perception of susceptibility and severity of COVID-19 disease showed significant association with willingness to work (*p* = 0.002 and 0.009, respectively). Lack of experience in a pandemic and fear about isolation or quarantine were also significantly associated with willingness to work (*p* = 0.002 and 0.003, respectively). HCWs with higher stress levels and those who were disappointed reported less willingness to work (Table 2).

**Table 2:** Health care workers' attitudes and factors related to willingness to work.

**Factors/attitude**	**Willing to work**		*P-*value^*^
	**Yes (%) 268 (75.1)**	**No (%)89 (24.9)**	
Stress from catching infection			0.007
Yes	222 (72.5)	84 (27.3)	
No	46 (90.2)	5 (9.8)	
Feeling safe			0.001
Yes	90 (87.4)	13 (12.6)	
No	78 (70.1)	76 (29.9)	
Fear from transmitting infection to family			0.27
Yes	243 (74.3)	84 (25.7)	
No	25 (83.3)	5 (16.7)	
Perceive susceptibility			0.002
Yes	220 (72.1)	85 (27.9)	
No	48 (92.3)	4 (7.7)	
Perceive severity of COVID-19			0.009
Yes	228 (72.8)	85 (27.2)	
No	40 (90.9)	4 (9.1)	
Lack of experience in such pandemic			0.002
Yes	199 (71.3)	80(28.7)	
No	69 (88.5)	9 (11.5)	
Fear from isolation/ quarantine			0.003
Yes	168 (70.3)	71 (29.7)	
No	100 (84.7)	18 (15.3)	
Availability of PPE			0.60
Yes	162 (76.1)	51 (23.9)	
No	106 (73.6)	38 (26.5)	
Stress			<0.001
Low	86 (90.5)	9 (9.5)	
High	182 (69.5)	80 (30.5)	
Feeling disappointed			0.048
Yes	144 (70.9)	59 (29.1)	
No	124 (80.5)	30 (19.5)	

* *Chi square test*.

Variables significantly associated with willingness to work were entered into a multivariable regression model in an enter mode manner. After controlling confounders, physicians and nurses reported significantly higher willingness to work than others (*p* = 0.004, Adj. OR = 3.5). Those with lower stress levels were two times more willing to work than higher stressed participants (*p* = 0.03). Additionally, willingness to work was significantly related to professional experience; HCWs with more than 10 years' experience were more willing to work than juniors. No significant associations with age, having children, quarantine or infection fear, perceiving of disease severity, and lack of experience existed (Table 3).

**Table 3:** Multivariable model of factors independently associated with willingness to work.

**Variable**	**SE**	*P*-value^*^	**Adjusted OR**	**95% CI**
Age				
<35 years^†^	0.43	0.8	1.1	0.5–2.5
≥35 years				
Work setting				
PHC	0.28	0.5	1.2	0.7–2.1
Hospital^†^				
Job title				
Physician				
Nurse	0.3	0.4	1.3	0.7–2.4
Others^†^ ^‡^	0.4	0.004	3.5	1.5–8.3
Experience				
<10 years^†^	0.4	0.03	2.6	1.1–6.1
≥10 Years				
Having children				
Yes^†^	0.4	0.5	0.8	0.4–1.5
No				
Stress				
Low	0.4	0.03	2.5	1.1–5.5
High^†^				
Quarantine fear				
Yes^†^	0.3	0.2	1.5	0.8–2.9
No				
Infection fear				
Yes^†^	0.6	0.1	2.6	0.8–8.4
No				
Perceived severity of COVID-19				
Yes^†^	0.6	0.1	1.7	0.7–3.9
No				
Lack of experience				
Yes^†^	0.4	0.2	1.5	0.8–2.9
No				

† Reference Group,

‡ lab and radiology technicians;

* *Significance level ≤ 0.5; OR, odds ratio; CI, confidence interval*.

### Qualitative Interviews

Fifteen HCWs were interviewed. The average age was 41 years (range 27–56), with more than half female (60%), and the occupation distribution was seven physicians, six nurses, and two other HCWs (lab and radiology technicians). Themes we focused on for this research include duty to work, perceived stressors, and issues related to the occupation. Each is presented.

#### Duty to Work

Participants universally felt a duty to work. One expressed a patriotic commitment “because I love Palestine.” The laboratory technician was altruistic, stating:

“*I knew no one will work in PCR lab with the highly contagious virus... so I volunteered to help. I felt it is my responsibility because I have the skills and also my personal responsibility to help people in my community.”*

While participants felt obliged to work and Ministry of Health (MOH) prohibited vacations, many knew colleagues who refused to work. One said, “Let me tell you there are no ethics in this pandemic.” Several were troubled by coworkers and supervisors who did not share the same sense of duty. A nurse said, “I have conflict with a physician because he refused to work with a patient.” A technologist stated, “Colleagues lacked professional ethics. Everyone wants to discharge himself from work and that caused a huge workload for the others.” As described by the technologist, some participants were frustrated with their colleagues, but a few described positive experiences. One said, “There was a huge sense of cooperation, we worked together as a team and supported each other.” Whether HCWs were in this “working together” or “felt alone and unappreciated,” all described incredible stress.

#### Perceived Stressors

A 33-year-old physician said, “This is the hardest experience in my life.” Most found the hours long and grueling, their duties taxing and at times beneath them such as contact tracing or physicians delivering food to quarantined patients. One physician complained, “My colleague and I worked alone to collect half of a random community sample for 1,500 in the district in 48 h. It was unfair, we were exploited.”

Working conditions felt unsafe due to inadequate and cheap PPE and no training. One reported, “Some colleagues were exposed to positive cases with no adequate PPE.” Another described the PPE “as cheap and not safe. When we were sprayed with water, it soaked through. The virus is much smaller than a water drop, so logically it was not safe.” Another explained, “We didn't have any orientation about this situation, no presentations or workshops or anything. Only YouTube videos oriented us what to do. that I had to find.” That included how to wear PPE, how to collect nasal swabs, etc. A female nurse said, “The lack of preparedness caused a huge workload, fear, and extreme floundering.”

In addition, many were frustrated by the lack of recognition for their efforts: “two months and not even verbal thanks.” In fact, MOH withheld pay. “Instead of reward, they [MOH] announced that 2 days will be discounted from our salary to support governmental actions.”

The challenge of how to work and meet family obligations was especially challenging for female HCWs who bore the brunt of children and elderly parents. A female physician said, “The biggest challenge as a female is the unavailability of a nursery as a result of the country lockdown. I don't know what to do with my kids.” A few needed accommodations due to personal health issues or family obligations such as caring for an ill parent. A female physician explained:

“*I approached the ministry officially asking about ‘A' shifts only because my father has a pulmonary embolism and I am the one responsible for looking after him and giving his medications... but they refused!”*

HCWs also described the emotional toll, including not seeing family, the fear of infecting family members, or getting sick themselves. A female physician explained, “Due to the quarantine and lockdown, I can't see my family and I miss their support.” Another who did go home said, “When I return home, I take off all my clothes at the door and do all the possible disinfection before entering to see my kids.”

Demonstrating the burden HCWs carried, one participant told the interviewer, “You covered all the issues hidden in my heart. Thank you for bringing them up.”

While the novel virus was a challenge around the globe and securing PPE and understanding about the diagnosis and management were evolving, some stressors might have been mitigated by better leadership. Participants reported limited support from supervisors. The lack of preparation and training is described above. Guidelines about who should not work due to health risks and arranging fair work distribution were largely missing. Supervisors “played favorites” and “there was no transparency.” Another explained that “duties were not distributed fairly, some were not asked to do fieldwork and only had to do prestigious work.” A female physician who had had cancer the year before said,

“*I thought I have to discharge myself from the duty to work in this pandemic, but when my physicians and my senior manager told me that this will not be accepted as an excuse to be discharged from duty, I cried a lot.”*

Her colleagues lobbied for her, and she was eventually dispensed from her direct care duties.

These leadership inadequacies contributed to the stressors outlined above. One HCW concluded: “It is our duty and obligation [to work]. But those with chronic or immunosuppressive disease should have been relieved from duty, but this was not the case here.”

#### The Occupation

The lockdown restricted travel and made it difficult for some to get to work or to see their families during periods of work. This was above and beyond the usual traffic patterns related to the occupation where checkpoints obstruct traffic flow and Palestinian access roads wind around Israeli freeways to and from settlements and Israeli cities. Normally, this adds substantial time to travel because only cars with certain license plates can use the Israeli roads. During the lockdown, this was even worse, and some found it easier to walk to work, but even that was difficult. One participant reported, “walking hours each way.” Walking was easier than driving because Palestinian forces closed roads for security reasons and checkpoints controlled by the Israeli Defense Forces had more erratic hours than usual, opening and closing without warning. Finally, the realities of the occupation force some Palestinians to work in Israel due to a lack of job opportunities in Palestine. This caused another layer of complication and potential COVID exposure that HCWs in those locations had to deal with. One explained:

“*After the agreement between the two governments (Palestinian and Israeli) to let the Palestinian workforce in Israel stay there for a one-month period, we were surprised that they returned back illegally from places other than the checkpoints provided by Israelis. This caused a huge challenge. We were waiting at the checkpoint 24/7 but most of the workers entered illegally supported by Israeli coverage. They infected their families which increased the work burden on us and challenged MOH capabilities.”*

The occupation added another layer of uncertainty and burden to the challenges of staying safe and caring for patients during the pandemic.

## Discussion

Frontline Palestinian HCWs faced extremely challenging work settings during the initial phase of the COVID-19 pandemic. Their ability to manage and cope with the huge work overload affected their willingness to work. The deontological and utilitarian approaches, judging actions as good or bad according to a clear set of rules, appeared to dominate health practice during the early months of the pandemic and raised many ethical dilemmas for our participants. In fact, most felt the duty to work but raised concerns about their safety, questioning the Hippocratic principles under which HCWs generally behave.

Almost one-fourth of Palestinian HCWs were unwilling to work during the pandemic. However, qualitative work demonstrates that unwillingness did not preclude HCWs from working. Attitudes about working seemed to be a continuum from a sense of duty, professionalism, and obligations to MOH and communities on one end and serious concerns about the high risk of personal safety on the other. During other pandemics, the more severe the pandemic, the higher HCW absenteeism and the less willingness to work (22). This further magnified the challenges in a country like Palestine, where limited resources, budget constraints, and conflict are ongoing realities.

The literature shows a wide range in outcomes (15), nevertheless, many studies demonstrated comparable results (23, 24). The debate on duty to care has been reported since the emergence of HIV/AIDS (25). Traditionally, it has been argued that physicians should have high standards of altruism and beneficence and hence have a duty to care for patients even at a risk to themselves (26). But during previous infection outbreaks, HCWs caring for sick people thought about dropping their work duties even though it was ethically unacceptable to them (14, 27). Additionally, a proactive approach, which explored the willingness to work in the case of infectious disease outbreak or bioterrorism among a large sample of American primary care physicians, revealed one fifth prevalence of unwillingness to work in such settings (28). As the pandemic has continued with additional peaks, pandemic fatigue has become more of a concern (29).

Fears of safety and security were evident in both our quantitative and qualitative data. PPE availability did not show a significant association with willingness to work, but interviews explored the quality and limited availability, which were of grave concern to many. Inadequate safety precautions increased Palestine HCWs' stress level and reflected on their willingness to work. While adequate PPE and evolving guidance may be difficult given the limited resources of MOH and the emerging understanding of the novel virus, better leadership despite the uncertainty might have mitigated some of the stressors.

Thought leaders on managing burnout in the health care workforce outlined the areas where US HCWs wanted support during the current pandemic: hear me, protect me, prepare me, support me, and care for me (30). This parallels much of what we heard from our participants, who were both stressed and disappointed, due to inadequate PPE, poor preparation and training, lack of guidelines about who should avoid exposure to the virus (age and health history), and limited efforts to create fair work assignments. Instead, many supervisors played favorites and were unwilling to accommodate family care needs, especially for women. In addition, for the most part, HCWs felt unappreciated and supervisors' efforts to build teams and a sense of mission in spite of uncertainty were missing. These are domains of good leadership. Research shows that good leadership is imperative in a pandemic (31) and even more important as the pandemic continues (30, 32). This is something MOH can and should address with their leadership teams in both hospital and ambulatory settings.

While childcare obligations were a reported barrier to HCWs' willingness to work in pandemics (22), our survey results (more than 55% female) showed that Palestinian HCWs were more willing to work if they had children. We may expect this finding in a conflict area like Palestine where tragedies and challenges at the political, economic, and social levels force people to struggle to provide for their families. Work may be considered an obligation to meet life's demands and to live with dignity. In fact, many interviewees expressed their duty to work as an obligation; some stated administration forced them to work and MOH forbid any type of vacations. However, the financial realities may be a hidden and unexpressed element.

While three quarters of the survey sample and all interviewees were willing to work, the stress and anxiety were high (72%), but those with less perception of stress and more professional experience were more willing to work. Emotional turmoil is mitigated by support, and institutional programs that address HCWs' mental health issues and focus on their psychosocial well-being to increase their resilience and reduce the magnitude of expected stress on quality of health care services are recommended by the World Health Organization (33, 34). Chinese efforts to address staff mental health needs during the current pandemic showed favorable results and helped HCWs improve the care they provided (35). Public health and policy makers should consider implementing this as we continue to struggle with COVID-19.

As an occupied territory, Palestine confronts COVID-19 from the perspective of the existing Israeli occupation, which weakens the Palestinian Authority's and the Palestinian people's ability to respond effectively to the deadly virus (36). COVID-19 does not distinguish borders, and the Palestinian and Israeli economies are intertwined. As many as 60,000 Palestinians are working in Israel and returning to their homes on a daily basis. While many conflict countries, in line with the current situation, have ceased fire and eased political tension, Israel's occupation has multiplied threats on Palestinians and aborted provisions to limit the transmission of COVID-19 as was expressed in our interviews.

Our study has many strengths. The sample size was large enough to focus on HCWs by profession and distinguish their responses based on place of work. The study took place during the beginning and the peak of cases of the outbreak in Palestine when the uncertainty and potential risks to self and family were the highest. The mixed design provides additional understanding, explanations, and interpretations of the quantitative findings.

The study, however, has cross-sectional study design limitations. The self-administered structure of the questionnaire may be prone to social desirability bias, as HCWs may elucidate positive responses to preserve their figures or as a result of potential perceived coercion from superiors. On the other hand, although the response rate for sample population (HCWs) is considered high, it leaves potential for non-response bias where non-respondents may have characteristics that vary from survey respondents. Besides, the fact that the research was performed in a public health emergency situation could also have limited the opportunity for the busiest and overburdened health workers to participate. The willingness to work among HWCs in Palestine may be either over or underestimated. Despite these realities, this study sheds light on the challenges of COVID-19 pandemic in a region with limited research to date.

In conclusion, Palestinian HCWs reported unwillingness to work amid the COVID-19 pandemic. Physicians and nurses were more likely to comply with a commitment to their professional ethics and the duty or obligation to work. However, stress levels and disappointment were high and could be mitigated in the future with better leadership, in spite of the uncertainty, and adding supports to address mental health and psychosocial challenges and enhance well-being. Finally, the political situation in Palestine creates budget constraints and fragmentation of the Palestinian Authority's response. Israel imposes further restrictions on the freedom of movement, and the lack of cooperation between Palestine and Israel further threatens the health security of Palestinians during this pandemic (36).

## Data Availability Statement

The raw data supporting the conclusions of this article will be made available by the authors, without undue reservation.

## Ethics Statement

The studies involving human participants were reviewed and approved by Al-Najah National University Institutional Review Board (IRB) Ref: F. Med June /20/7. The patients/participants provided their written informed consent to participate in this study.

## Author Contributions

BM contributed to the literature search, conceptualization and study design, development of questionnaire, data collection, analysis, data interpretation, and manuscript writing. ZN contributed to the conceptualization and study design, development of questionnaire, analysis, data interpretation, and manuscript writing. TZ contributed to the development of questionnaire, data interpretation, and manuscript writing. All authors have seen and approved the final version of the manuscript for submission.

## Conflict of Interest

The authors declare that the research was conducted in the absence of any commercial or financial relationships that could be construed as a potential conflict of interest.
