# Supplementary material for: Mixed Method Study to Explore Ethical Dilemmas and Health Care Workers' Willingness to Work Amid COVID-19 Pandemic in Palestine
Source: Front Med (Lausanne). 2021 Jan 5;7:576820. doi: 10.3389/fmed.2020.576820 (PMC7813812; doi:10.3389/fmed.2020.576820)
Supplement: Supplementary file 1 [file Data_Sheet_1.docx]

**Mixed Method Study to Explore Ethical Dilemmas and Health Care Workers' Willingness to Work Amidst COVID-19 Pandemic in Palestine**

**Dear Colleague,**

In the light of these extraordinary circumstances, which have demonstrated the importance of your role as a health worker in fighting and restricting the spread of the Coronavirus and securing the health of your community, we thank you and appreciate your efforts.

This link includes an online questionnaire, which **aims** to measure the psychological pressures experienced by health workers in Palestine during their work in COVID 19 pandemic, possible associated factors, and coping strategies.

After you agree to participate, we ask you to answer the following questions in an open and honest manner for the purposes of scientific research. We hope survey like this that can help us find better ways to manage this crisis and others in the future.

**The questionnaire takes about 5 minutes**. Your participation is **voluntary**and **anonymous**.

This study does not include any participant-identifying information. Your answers will not be associated with who you are or where you work. Your responses will be treated strictly confidential and will only be used for scientific research purposes.

We are grateful for your cooperation and wish you continued health and wellness.

You can talk to the study team about any questions, concerns, or complaints you have about this study.

**Contact the study doctor(s)**

**Dr. Beesan Maraqa - Dr. Zaher Nazzal**

|  |  | **Section I** |
| --- | --- | --- |
|  |  | **Demographic characteristics** |
|  | ............................... | Place of work (District) |
|  | ………………………. | Job title |
|  | ................................ | Experience (years) |
|  | ................................ | Age |
| 🞏Hospital | 🞏Primary health care | Work Setting |
| 🞏Female | 🞏Male | Sex |
| 🞏Married  🞏 Widow | 🞏Single  🞏Divorced | Marital status |
| 🞏No | 🞏Yes | Do you have children |
| 🞏No | 🞏Yes | Did you live with your family at the time of the outbreak |
| 🞏No | 🞏Yes | Have you dealt with a COVID 19 case |
| 🞏No | 🞏Yes | Are you willing to work during the pandemic |

| **Section II** | | | | | | |
| --- | --- | --- | --- | --- | --- | --- |
| **No** | **Yes** | | | | | **Please respond to the following statements** |
|  | **Very much** | **Much** | **Average** | **Little** | **Very little** |  |
|  |  |  |  |  |  | I feel stressed because of the COVID-19 outbreak |
|  |  |  |  |  |  | I'm afraid of getting the COVID-19 infection |
|  |  |  |  |  |  | I feel safe and that things are under control |
|  |  |  |  |  |  | I fear transmitting COVID-19 to my family |
|  |  |  |  |  |  | Being a health professional raises my COVID-19 threat |
|  |  |  |  |  |  | COVID-19 is a real threat since no treatment has been available so far. |
|  |  |  |  |  |  | COVID-19 is a real concern because we lack the experience to cope with such a pandemic. |
|  |  |  |  |  |  | I felt disappointed while working on this outbreak |
|  |  |  |  |  |  | I am afraid to be isolated and/or quarantined |
|  |  |  |  |  |  | I think we've got the PPE through the Pandemic |
