# Supplementary material for: Mixed Method Study to Explore Ethical Dilemmas and Health Care Workers' Willingness to Work Amid COVID-19 Pandemic in Palestine
Source: Front Med (Lausanne). 2021 Jan 5;7:576820. doi: 10.3389/fmed.2020.576820 (PMC7813812; doi:10.3389/fmed.2020.576820)
Supplement: Supplementary file 2 [file Data_Sheet_2.docx]

**Interview Guide**

**Mixed Method Study to Explore Ethical Dilemmas and Health Care Workers' Willingness to Work Amidst COVID-19 Pandemic in Palestine**

*Statement about anonymity, confidentiality of responses, and data protection methods.*

Q1 What are your thoughts regarding your duty to work during COVID-19 pandemic:

- What motivates you to work during this pandemic? What motivates your colleagues?
- How do you perceive your relationships with your colleagues?

Probe :

- professional ethics
- employee responsibility
- duty to help
- human nature
- spiritual obligation
- not letting patients or colleagues down
- if one is able to work is it right to take sick leave
- if the Ministry doesn’t forbid vacations; will you apply for vacation?

Q2 What are the top or biggest barriers towards working in this pandemic for you?

Probe:

- child care and family obligations
- fear from transmitting infection to family
- lock down, community quarantine and remote work place
- training
- perception of risk to oneself
- Israeli occupation and not controlling border/barrier crossings

Q3 Tell me what bothers you most in this pandemic? What do you see as most bothering your colleagues?

Probe:

- Long working hours
- No rewards
- No financial compensation
- Cheap PPE
- No guidance, information
- No transparency

Q4 How do you perceive your occupational risk for working in COVID-19 pandemic

Probe:

- No vaccine
- Crowded work place
- Different work duty
